# Thank you, Professor Heidi Pfeiffer!

**DOI:** 10.1007/s00414-023-03145-4

**Published:** 2023-12-05

**Authors:** Andreas Schmeling

**Affiliations:** Institute of Legal Medicine, University Hospital Münster, Münster, Germany

Dear Colleagues,

I am writing to inform you that Prof. Heidi Pfeiffer has recently announced her decision to step down from her role as Editor-in-Chief of the *International Journal of Legal Medicine*. Since 2009, she has admirably led the journal’s Editorial board. During her 15-year tenure, the journal has developed very successfully both quantitatively and qualitatively. The number of articles published annually has more than doubled. In terms of impact, the *International Journal of Legal Medicine* has maintained its position as one of the world’s leading medicolegal journals.

This is an opportune moment to express our gratitude for her dedicated and outstanding contributions, her unwavering and supportive guidance to the Editorial board members and reviewers, and her acute leadership.

To ensure the seamless continuation of the journal’s excellence, Prof. Tony Fracasso will succeed Prof. Heidi Pfeiffer as the new Editor-in-Chief, sharing the responsibilities alongside me.

Acknowledging the growing specialization within legal medicine and its subdisciplines, we have chosen to enlist the aid of distinguished specialists to serve as associate editors:

- Prof. Jens Amendt for the section «Entomology»
- Prof. Volker Auwärter for the section «Toxicology»
- Prof. Eugenia Cunha for the section «Anthropology»
- Prof. Sabine Lutz-Bonengel for the section «Genetics»

We firmly believe that this revamped editorial team is well-equipped to effectively lead the journal in the forthcoming years.

Warm regards,

Andreas Schmeling.

